# Toward Low-Cost Affinity Reagents: Lyophilized Yeast-scFv Probes Specific for Pathogen Antigens

**DOI:** 10.1371/journal.pone.0032042

**Published:** 2012-02-20

**Authors:** Sean A. Gray, Kris M. Weigel, Ibne K. M. Ali, Annie A. Lakey, Jeremy Capalungan, Gonzalo J. Domingo, Gerard A. Cangelosi

**Affiliations:** 1 Seattle Biomedical Research Institute, Seattle, Washington, United States of America; 2 Division of Infectious Disease and International Health, University of Virginia Health System, Charlottesville, Virginia, United States of America; 3 PATH, Seattle, Washington, United States of America; James Cook University, Australia

## Abstract

The generation of affinity reagents, usually monoclonal antibodies, remains a critical bottleneck in biomedical research and diagnostic test development. Recombinant antibody-like proteins such as scFv have yet to replace traditional monoclonal antibodies in antigen detection applications, in large part because of poor performance of scFv in solution. To address this limitation, we have developed assays that use whole yeast cells expressing scFv on their surfaces (yeast-scFv) in place of soluble purified scFv or traditional monoclonal antibodies. In this study, a nonimmune library of human scFv displayed on the surfaces of yeast cells was screened for clones that bind to recombinant cyst proteins of *Entamoeba histolytica*, an enteric pathogen of humans. Selected yeast-scFv clones were stabilized by lyophilization and used in detection assay formats in which the yeast-scFv served as solid support-bound monoclonal antibodies. Specific binding of antigen to the yeast-scFv was detected by staining with rabbit polyclonal antibodies. In flow cytometry-based assays, lyophilized yeast-scFv reagents retained full binding activity and specificity for their cognate antigens after 4 weeks of storage at room temperature in the absence of desiccants or stabilizers. Because flow cytometry is not available to all potential assay users, an immunofluorescence assay was also developed that detects antigen with similar sensitivity and specificity. Antigen-specific whole-cell yeast-scFv reagents can be selected from nonimmune libraries in 2–3 weeks, produced in vast quantities, and packaged in lyophilized form for extended shelf life. Lyophilized yeast-scFv show promise as low cost, renewable alternatives to monoclonal antibodies for diagnosis and research.

## Introduction

Defined as antibody (Ab)-like molecules that bind to specific antigens, affinity reagents are critical tools in biomedical research, biomarker discovery, and diagnostic testing. Generation of monoclonal antibodies (MAbs) by traditional methods, typically by the mouse hybridoma route, is a significant bottleneck in biomedical research and development. MAbs in diagnostic tests often require significant optimization before being usable for diagnostic assays, and licensing costs can exceed all other test costs combined. Cheaper and more available affinity reagents would greatly facilitate biomedical research and empower developers of new diagnostic tests.

Recombinant antibody-like molecules such as single chain Fragment variable (scFv) and fragment antigen binding (Fab) are potentially appealing alternatives to MAbs. Libraries of these molecules have been displayed on the surfaces of organisms including *E. coli*, phages, yeast, and on ribosomes. Although these methods have existed for many years, few such fragments have proven useful as molecular probes in diagnostic tests. Methods for rapidly selecting antigen-binding yeast-displayed scFv clones were described nearly ten years ago [1]. Yeast display libraries express scFv on the surface of *Saccharomyces cerevisiae* cells. Using a combination of magnetic bead enrichment and fluorescent-activated cell sorting (FACS), yeast clones that bind specifically to antigens can be selected from naïve libraries in 2–3 weeks. This selection process is much faster and less expensive than the mouse hybridoma route. However, scFv selected by this method rarely perform well when secreted into solution. Like natural antibodies, yeast-displayed scFv are products of selection, in this case for activity in the environment of a yeast cell surface. Hence, many yeast-displayed scFv perform poorly in other environments, especially when secreted. Although functional soluble scFv have been reported [2], [3], [4], [5], [6], [7], [8], in practice the great majority of scFv culled from yeast display libraries have exhibited unsatisfactory activity in standard immunoassay formats.

One solution to this problem is to forgo the generation of soluble scFv, and instead use the yeast-displayed scFv (yeast-scFv) directly as detection reagents. In this strategy, the yeast cell provides a surface on which to present the scFv, which is similar in nature to a plastic or ceramic bead particle that has been coupled to an antibody. An example of this strategy was reported by Cho *et al*, who used yeast-displayed scFv to immunoprecipitate (IP) brain endothelial proteins from a complex cell lysate [9]. In this method, bound proteins were eluted from the yeast cell surface by mild reduction, and the eluted proteins subjected to mass spectrometry to reveal their identity [this technique was termed Yeast Display Immunoprecipitation (YDIP) [9]]. Another example is a competitive-inhibition flow cytometry (CIFC) assay [7]. CIFC is an indirect assay in which antigen capture is detected and quantified by competitive inhibition of yeast-scFv binding to fluorescently labeled antigen.

A disadvantage of both YDIP and CIFC is that yeast-scFv probes must be cultured and induced to express scFv before each assay. In addition, YDIP requires mass spectrometry to detect captured antigens, a potentially cumbersome procedure requiring significant laboratory instrumentation. CIFC has its own limitations, being an indirect format that is susceptible to false-positive results due to non-specific inhibition of fluorescent antigen binding.

The present study sought to overcome these limitations to yield truly practical, direct yeast-scFv assay formats. Induced yeast-scFv probes were lyophilized to yield stable reagents that did not require cultivation or special storage conditions, and were immediately usable following rehydration. In addition, simple direct assay formats were developed that used yeast-scFv reagents in combination with basic laboratory resources.

To evaluate these methods, we isolated yeast-scFv probes that bound specifically to putative cyst proteins of the parasite *Entamoeba histolytica*, the causative agent of intestinal amoebiasis in developing countries [10], [11]. These proteins were previously shown by gene expression profiling analyses to be up-regulated in *E. histolytica* cysts [12], thereby making them candidate stool biomarkers of *E. histolytica* infection. The study demonstrates a new type of renewable affinity reagent that can be selected from non-immune yeast display libraries in 2–3 weeks, produced in vast quantities by yeast culture, and packaged in lyophilized form for extended shelf life.

## Results

### Library selections

Four *E. histolytica* proteins were selected for this study on the basis of their elevated mRNA expression in the cyst stage relative to the trophozoite stage of the parasite [12]. Fragments of each protein were selected and cloned to minimize sequence overlap with homologs from the commensal species *E. dispar* and *E. moshkovskii*. These species are closely related to *E. histolytica* and are often found in stool samples, but are not pathogenic to humans [13], [14], [15]. The protein fragments, which ranged in size from 12 to 34 kDa, were cloned for expression in *E. coli* (Table 1). One of the proteins, *Eh*Jacob1, is expressed at approximately 1290 fold higher mRNA level in the cyst form relative to the trophozoite form of the parasite [12]. In early encysting *E. invadens* cells (a model parasite for studying encystation in *E. histolytica*), Jacob proteins appear on the surface of encysting parasite and are abundant in the walls of mature cysts [16], [17]. Three chromodomain-containing proteins (EHI_142000, EHI_115350 and EHI_000780; hereafter termed 142, 350, and 780, respectively) were also selected because chromodomain proteins fulfill stage-specific functions in other systems [18], [19]. Additionally, 780 was detected in 2 out of 5 *E. histolytica* cyst samples that were subjected to shotgun proteomic analysis (Ali *et al.*, unpublished).

**Table 1:** *E. histolytica* proteins used in this study.

Protein	EHI #	NCBI Accession	Total AA^1^	AA Expressed	MW (kDa)^2^	Annotation	Yeast-scFv probe
Jacob	EHI_044500	XP_657246	574	323	34	Cyst wall-specific glycoprotein Jacob1	*Jacob-A11*
780	EHI_000780	XP_653178	1641	148	19	Chromodomain-helicase-DNA-binding protein	*780-23*
350	EHI_115350	XP_649984	1247	138	17	Chromodomain-helicase-DNA-binding protein	*350-E2* *350-12*
142	EHI_142000	XP_654629	414	117	12	Histone acetyltransferase	none

1 AA, number of amino acids.

2 Calculated molecular weight of expressed protein fragments.

The Wittrup library of human nonimmune scFv antibodies displayed on the surface of yeast (gift of K. Dane Wittrup, Massachusetts Institute of Technology) was used to isolate scFv that bind to *E. histolytica* proteins. Following magnetic and FACS selections, sorted clones were tested for binding to cognate antigen as well as for specificity. To determine specificity, antigen-binding clones were tested for non-binding to a minimum of four additional *E. histolytica*-derived non-cognate antigens.

A total of 48 selected clones were tested to confirm binding to a biotinylated 350 fragment (350-biotin). Of these, 30 clones specifically bound the antigen. However, *Bst*N1 fingerprint analysis followed by DNA sequencing revealed that only two clones, designated *350-12* and *350-E2*, were isolated. Clone *350-E2* was isolated more than 20 times from multiple sorts with this antigen. Further characterization of these clones revealed that *350-12* bound only biotinylated-350, and failed to bind unlabeled 350, while clone *350-E2* bound both labeled and unlabeled 350.

For the 780 antigen, eight unique clones were isolated from 48 selected clones. All 8 of these clones bound biotinylated-780, and none of them bound unlabeled 780 antigen. Biotinylation may have altered antigen structure such that unlabeled antigen was no longer recognized. One of the unique clones, designated *780-23*, bound the 780-biotin antigen at a moderately high affinity of ∼1 nM as determined by using a flow cytometric assay described previously [6], [7]. This clone was chosen for lyophilization studies.

Selections with the Jacob antigen generated several unique clones. One clone, designated *Jacob-A11*, bound to both biotinylated and unlabeled Jacob antigen. The affinity was determined to be 43 nM and this clone was selected for further studies. The 142 fragment generated a panel of yeast scFv that exhibited good binding. However, all of these scFv exhibited non-specific binding to at least one of the four non-cognate control proteins employed for the specificity analysis. No clones were isolated that bound specifically to 142.

In summary, yeast-scFv clones were isolated that bound specifically to 3 out of 4 protein fragments examined. The following 4 yeast-scFv clones that bound specifically to putative *E. histolytica* cyst proteins were chosen for assay development: clones *350-E2* and *350-12* which bound the 350 antigen; clone *780-23* which bound the biotinylated 780 antigen, and clone *Jacob-A11* which bound the Jacob antigen.

### Yeast-scFv lyophilization and assay formats

**Figure 1:**
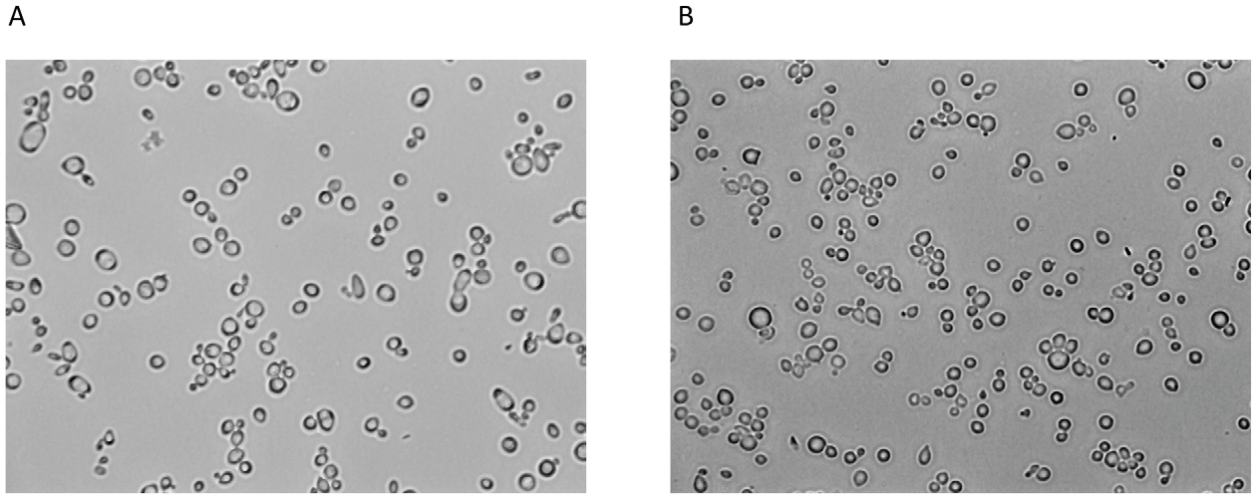
Morphologies of fresh and lyophilized yeast. Ten microliters of fresh induced (Panel A) or lyophilized and rehydrated (Panel B) yeast-scFv clone *350-E2* was analyzed by light microscopy at 40× magnification.

Selected yeast clones were induced to express scFv and antigen binding was confirmed by flow cytometry. The yeast cells were then lyophilized as described in *Materials and Methods*. Yeast that were lyophilized and rehydrated in assay wash buffer appeared morphologically similar to fresh, pre-lyophilized yeast (Figure 1). Both pre-lyophilized yeast and reconstituted yeast appeared rounded and uniform with approximately half of the yeast in the process of budding. By flow cytometry, forward scatter (FSC) versus side scatter (SSC) plots indicated slight differences between fresh and lyophilized yeast. Specifically, yeast that had been lyophilized and rehydrated shifted to the left on SSC histograms relative to fresh yeast, while remaining largely unchanged on FSC histograms. This suggests that lyophilization and rehydration reduces the complexity and granularity of yeast-scFv cells without significantly changing their size.

**Figure 2:**
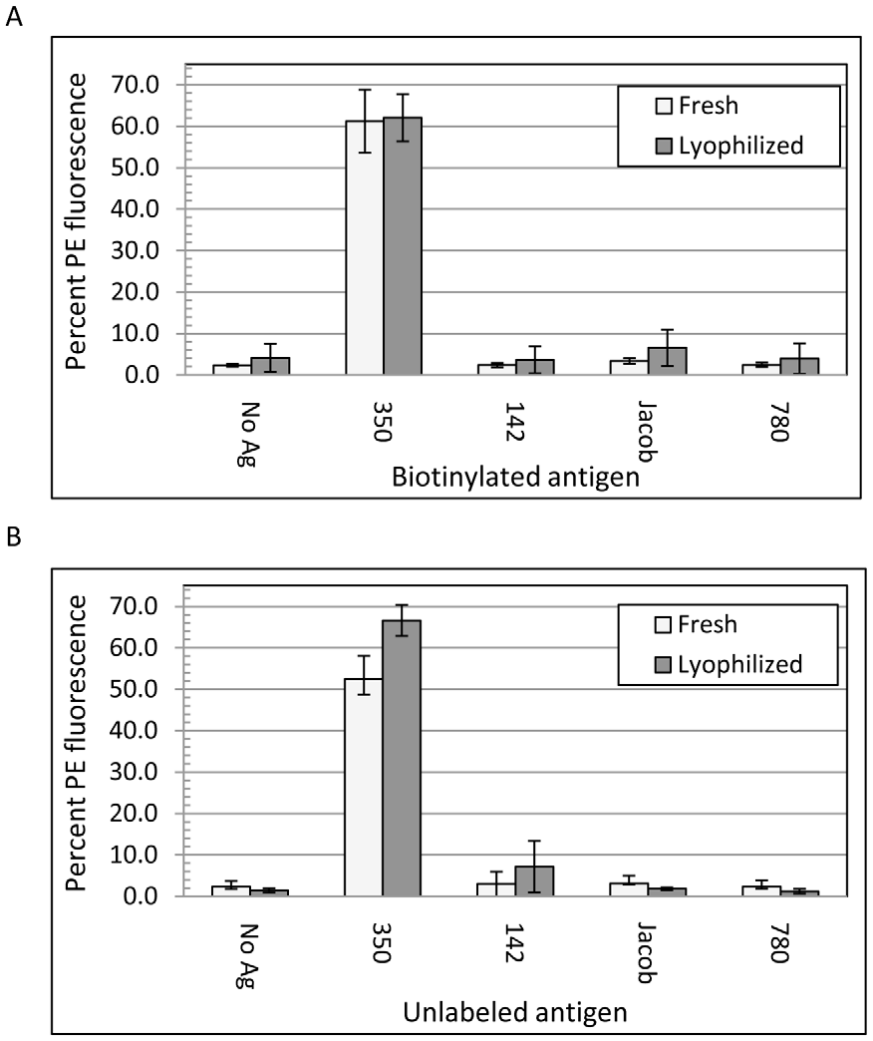
Pre- and post-lyophilization antigen binding and specificity of clone *350-E2*. Fresh, non-lyophilized *350-E2* yeast and lyophilized, rehydrated *350-E2* yeast were split into 10 aliquots. In Panel A, each aliquot received either no antigen, or 100 nM each of 350, 142, Jacob, or 780. Biotinylated antigens were used in every case, and antigen binding was detected by incubating with SA-PE. Panel B was performed as in Panel A, except that non-biotinylated antigens were used and antigen binding was detected by incubation with rabbit-anti-350 antiserum followed by GaR-PE. The percent of PE-positive yeast was determined by gating the PE fluorescence relative to the “no antigen” control. Values are means of three experiments plus or minus the standard deviation. The light grey bars indicate binding of the fresh yeast to each antigen, and the dark grey bars indicate binding of the lyophilized yeast to the antigens.

Lyophilized yeast-scFv probes were used in three antigen detection assay formats. In one format, used primarily for assay development purposes, biotinylated antigens were captured by yeast-scFv and bound antigen was then detected by staining with streptavidin conjugated to phycoerythrin (SA-PE) followed by flow cytometric analysis (results reported in Figure 2A). In a second, more broadly useful format, unlabeled antigens were incubated with yeast-scFv and their capture was detected by incubating the complexes with rabbit polyclonal antiserum raised against the antigen. Figure 2B shows the results of rehydrated yeast-*350-E2* binding to unlabeled 350 antigen. The antigen-rabbit polyclonal antibody complex was further detected using a phycoerythrin-conjugated goat-anti-rabbit MAb (GaR-PE) and analyzed by flow cytometry. The rabbit polyclonal antiserum was not affinity purified and therefore was unlikely to be fully specific to the cognate antigens. However, when such antibodies were used in sandwich assays in combination with monoclonal yeast-scFv probes, the combined assays were expected to be specific. In a third assay format, a sandwich assay incorporating yeast-scFv capture probes, polyclonal rabbit antibody signal probes and GaR-FITC detection probes were combined with an immunofluorescence (IFA) microscopy readout rather than flow cytometry.

In the flow cytometry-based assays, binding of antigen was quantified as the percent of PE-positive yeast relative to the no antigen control. In most such assays, positive binding resulted in PE-fluorescence of approximately 45–65% of yeast cells.

Lyophilization and reconstitution had little effect on binding of yeast-scFv to antigens. For example, freshly grown yeast-scFv *350-E2* exhibited approximately 53% binding to both unlabeled (Figure 2B) and biotinylated 350 antigen (Figure 2A) prior to lyophilization, and approximately 64% binding after lyophilization and reconstitution. The same probe exhibited only <6% binding to three non-cognate antigens (142, Jacob, and 780). This specificity did not change with lyophilization and rehydration, although slightly greater background binding to biotinylated non-cognate antigens was observed in lyophilized yeast. Specifically, fresh yeast exhibited 0.16–0.27% binding to biotinylated non-cognate antigens while lyophilized yeast exhibited 3.27–5.15% binding to these antigens. When unlabeled antigens were detected using rabbit polyclonal antibody followed by GaR-PE in place of SA-PE, both fresh and lyophilized yeast exhibited similar background binding levels. Therefore, it is likely that the SA-PE reagent is responsible for the non-specific binding seen in Figure 2A. In addition to clone *350-E2* (Figure 2), full activity following lyophilization was observed in 8 additional, distinct yeast-scFv clones specific to 3 different antigens (Figure S1). No clone examined failed to bind its cognate antigen following lyophilization. Therefore, antigen-specific binding after lyophilization appears to be a frequent property of yeast-scFv reagents.

While appearing healthy microscopically and retaining scFv expression and antigen binding, the lyophilized yeast did not retain viability under the lyophilization conditions used. Attempts to culture rehydrated cells demonstrated complete loss of viability.

### Lower limit of detection (LLD) of lyophilized yeast-scFv probes

**Figure 3:**
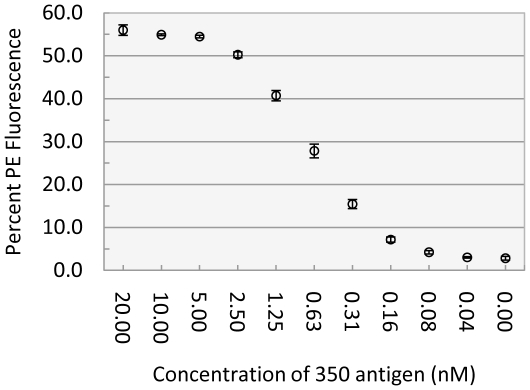
Lower limit of detection of 350 antigen by lyophilized yeast-scFv clone *350-E2*. Lyophilized *350-E2* yeast cells were reconstituted in 1 mL YWB and split into 11 equal aliquots. Each aliquot was incubated with 10 concentrations of 350-biotin antigen ranging from 20 nM to 0.04 nM (plus the 0 nM control) in twofold serial dilutions in 2.5 mL (total volume) YWB. Bound 350 antigen was detected by incubating with a rabbit anti-350 antiserum followed by goat-anti-rabbit-phycoerythrin (GaR-PE) detection. Samples were analyzed by flow cytometry. The entire experiment was repeated three independent times, and each replicate was performed with an individually grown, induced, and lyophilized *350-E2* clone. Each data point shows the average mean percent PE-positive yeast plus and minus the standard deviation.

To quantify the sensitivity of antigen detection by yeast-scFv reagents, we tested the *350-E2* probe against 10 concentrations of 350 antigen ranging from 20 nM to 0.04 nM in twofold serial dilutions, each in a volume of 2.5 mL. Bound 350 antigen was detected by incubation with rabbit anti-350 antiserum followed by GaR-PE and analyed by flow cytometry. As shown in Figure 3, the LLD was approximately 310 picomolar, comparable to an ELISA assay using a typical monoclonal antibody. The LLD was determined as the concentration of antigen resulting in binding was two standard deviations above the “no antigen” negative control.

### Immunofluorescence microscopy assay using yeast-scFv probes

**Figure 4:**
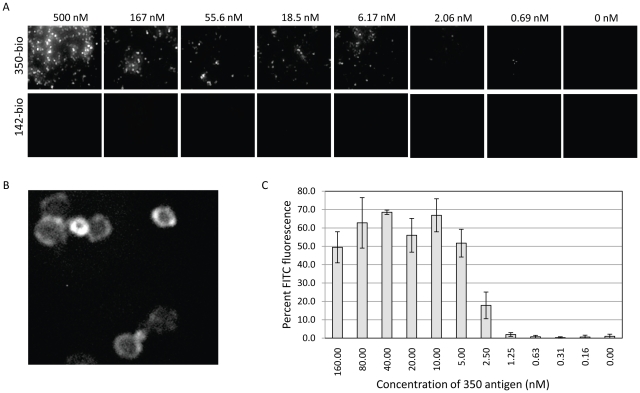
Immunofluorescence assay using lyophilized yeast-scFv reagents. Panel A shows lyophilized, reconstituted *350-E2* yeast binding to cognate 350 antigen (upper row) or non-cognate 142 (lower row) in concentrations ranging from 500 nM down to 0.69 nM in threefold serial dilutions. Bound antigen was detected by incubating with a rabbit anti-350 antiserum followed by GaR-FITC detection. Stained yeast were viewed at 40× magnification. Panel B is the 500 nM concentration of 350-biotin binding seen in Panel A, but photographed at 90× magnification and digitally zoomed 3× to visualize staining patterns. Panel C shows a graph of a second experiment in which 350-biotin was tested in triplicate using 11 concentrations ranging from 160 nM down to 0.14 nM in twofold serial dilutions. For each concentration, 3 fields were photographed twice, first in visible mode, and then in FITC mode. The percent of FITC-positive yeast were calculated and graphed relative to the concentration of 350 antigen.

The flow cytometry-based assays are sensitive and specific; however, flow cytometry capabilities are not available to all potential users of yeast-scFv probes. Therefore, we developed an alternative immunofluorescence microscopy assay (IFA) using lyophilized yeast-scFv. To evaluate the IFA, the reconstituted yeast-scFv (*350-E2* in Figure 4) were incubated with antigen in a concentration series ranging from 500 nM to 0.69 nM in threefold dilutions. Antigen binding was detected by staining with rabbit anti-350 antiserum followed by GaR-FITC, and quantified by fluorescence microscopy and computer-assisted image analysis as described in Materials and Methods. As shown in Figure 4A, FITC fluorescence in the presence of the cognate 350 antigen was clearly visible relative to a non-cognate control antigen (142). Under increased magnification (Figure 4B) the majority of the fluorescent staining appeared localized to the perimeter of the yeast-scFv particles, consistent with staining of the cell wall-associated scFv. By eye, the LLD of the IFA format was 6.17 nM (Figure 4A). To better quantify the LLD, we imaged three separate, randomly chosen fields first by visible light and then with FITC, to determine the percentage of total yeast-scFv particles that exhibited antigen binding above a threshold value (Figure 4C). By this approach, a LLD of 5.0-2.5 nM was observed. Although somewhat less sensitive than the flow cytometry assays, the immunofluorescence microscopy assay delivers useful sensitivity without requiring flow cytometry.

### Stability of lyophilized yeast-scFv

**Figure 5:**
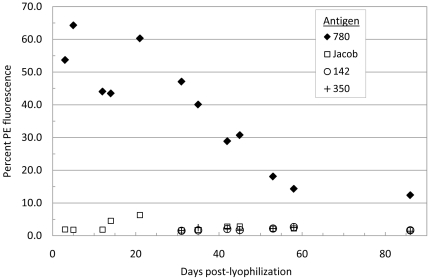
Lyophilization time course assay with yeast-scFv clone 780-23. One aliquot of lyophilized yeast was removed at intervals over the course of 86 days and stained first for c-*myc*-FITC expression followed by staining with cognate 780-biotin or three non-cognate antigens (Jacob-biotin, 350-biotin, or 142-biotin plus a no antigen control). Following detection of antigen binding with SA-PE, the percent of PE-positive yeast were determined using the no antigen stain as a gating reference. The graph depicts the percent positive yeast (*y*-axis) at each of the time points tested (*x*-axis) for each of the four antigens. The assay was terminated when binding to cognate 780-biotin antigen had dropped below 15%.

The stability of lyophilized yeast-scFv was assessed by using clone *780-23* which is specific for 780-biotin antigen (Figure 5). For each time point, an aliquot of lyophilized *780-23* yeast was rehydrated, stained for scFv expression via the anti-c-*myc* tag, and then split into smaller aliquots. One aliquot was incubated with 100 nM 780-biotin (cognate antigen) and other aliquots were incubated with 100 nM of three non-cognate antigens (Jacob-biotin, 142-biotin, and 350-biotin). Jacob-biotin was used throughout the experiment, while the other non-cognate antigens were used only at later time points. After washing, the bound antigen was detected by the addition of SA-PE. An aliquot of unstained yeast was also analyzed as a negative control. As seen in Figures 2 and 3, antigen binding was quantified using flow cytometry to measure the percentage of yeast cells that expressed scFv (FITC on the x-axis) and bound to biotinylated antigen (PE on the *y*-axis). Binding activity was stable for at least 30 days, with decay in the PE fluorescence starting around day 35. Binding to non-cognate antigens was low throughout the experiment (Figure 5).

Similar time course assays were carried out using clone *350-E2*, and both resulted in very similar trends. In this assay, binding to cognate antigen (350-biotin) remained constant (between 40% and 60%) for ≥30 days post lyophilization, while binding to a non-cognate control antigen (780-biotin) was low throughout the experiment (Figure S2).

## Discussion

Affinity reagents are critical limiting factors in biomedical research and diagnostics. Our goal is to design faster, cheaper, and more versatile methods for generating new affinity reagents. Yeast display libraries of human scFv have been in use for more than 10 years and are well established for rapid *in vitro* antibody isolation. However, the use of scFv culled from these libraries has been limited by the slow and inefficient conversion of yeast-displayed scFv to soluble, secreted antibodies that can be incorporated into diagnostic tests. As a result, yeast display and related methods have not supplanted traditional methods for generating monoclonal antibodies.

To address this challenge, we pursued an alternative model for yeast-scFv generation and application. We hypothesized that scFv probes culled from yeast-display libraries would function best if maintained in the environment in which they were selected, namely tethered by Aga1-Aga2 linkages to yeast cell walls. Accordingly, we developed methods to use yeast-scFv directly for antigen detection, without requiring secretion of scFv in soluble form. If enabled, the end-use detection reagents could be generated in two to three weeks, much faster than MAb generation.

We selected scFv that bound to cyst proteins derived from the human pathogen *E. histolytica*. Four proteins were selected for this study based on microarray analysis that indicated elevated mRNA levels in cysts relative to trophozoites. The proteins were produced recombinantly in *E. coli* and then biotinylated to perform selections for yeast-displayed scFv Abs. Only one of the four proteins (142) failed to yield specific yeast clones. While multiple clones were selected to each of the other three proteins, the majority of clones bound only to the biotinylated form of the proteins. We speculate that biotinylation may alter protein folding, exposing new epitopes not seen in the native protein. The selected yeast-scFv clones bound the biotinylated form of the protein, and not to the biotin entity itself, as indicated by the fact that they did not bind to biotinylated non-cognate control antigens. One clone, *Jacob-A11*, bound both unlabeled and Jacob-biotin protein, however it bound much better to Jacob-biotin. Only two unique clones were isolated that bound 350 antigen. This may be because the 350 protein fragment used for selections was small (∼18 kDa) and therefore may have presented a small number of epitopes. One of these clones, *350-E2*, bound labeled and unlabeled 350 antigen equally well and was isolated more than 20 times from multiple independent selections. This clone was used for most of the subsequent work.

A major goal of the study was to determine whether whole yeast displaying scFv could be preserved by lyophilization. Yeast clones were induced to express scFv on their surfaces, and then lyophilized in single use aliquots. Microscopically, lyophilized/reconstituted yeast were indistinguishable from freshly induced yeast. By flow cytometry the rehydrated yeast exhibited much lower side scatter fluorescence, suggesting that they lacked the complexity/granularity of fresh yeast. The lyophilized yeast did not form colonies when plated on SDCAA medium (synthetic dextrose medium with casamino acids deficient in histidine, uracil, and tryptophan). The loss of viability and shift in side scatter fluorescence may be a result of our simple lyophilization protocol, which did not use cryoprotectants. Our goal was not to maintain viability, but rather to maintain the specific antigen binding activities of the surface-displayed scFv.

In flow cytometric assays, clones maintained binding to cognate antigen that was similar to that of freshly induced yeast. Lyophilization did not increase non-specific binding to non-cognate antigens. Lyophilized yeast-scFv probe *350-E2* bound to its cognate antigen with a lower limit of detection (LLD) of less than 1 nM. This LLD approaches that of a typical ELISA assay using a monoclonal IgG antibody. In addition to flow cytometry, diverse alternative read-outs can be used including the IFA described here which detected 350 antigen with an LLD in the range of 2.5–6.17 nM.

The lyophilized yeast were stored at room temperature, reconstituted in simple buffers (typically PBS or PBS+5% BSA), and were immediately usable following rehydration. Although stabilizers such as lyophilization buffers, nitrogen gas, oxygen absorbers, and desiccants were not used, all three yeast-scFv clones examined maintained stable binding for at least 30 days. Longer shelf lives may be possible when yeast-scFv are stored with cooling or desiccants.

Lyophilization of yeast-scFv facilitates storage and shipment. Moreover, it abrogates the need for overnight growth of yeast in SDCAA medium, followed by overnight induction of scFv expression in SG/RCAA media, as well as the need to perform quality control (QC) analysis of each induction. With lyophilized yeast cultures, the QC is performed just once immediately prior to lyophilization. A typical flow cytometry assay, such in Figure 2, requires approximately 1e7 yeast. With lyophilization, liter-scale batches of induced yeast (at a concentration of 2-4e7 yeast per mL) can be stored in single-use aliquots of 1e7 yeast. This means that a single 1-liter culture of induced yeast-scFv can yield enough material for up to 4000 tests at a cost of a few pennies per test.

Most of the assays describe herein utilized rabbit polyclonal antisera for secondary detection of unlabeled antigens. We have also used polyclonal mouse and chicken antisera for this purpose. The use of polyclonal detection antibodies extends the time required to generate functional yeast-scFv-based antigen detection assays. However, polyclonal detection antibodies do not need to be affinity-purified, because assay specificity is conferred by the yeast-scFv, which are monoclonal. The only requirements for detection antibodies are 1) that they bind to antigens of interest, and 2) that they don't cross-react with yeast-scFv in the absence of antigen. Thus, polyclonal detection antibodies are inexpensive and easy reagents to prepare.

In summary, whole-cell yeast-scFv affinity reagents can be rapidly selected and prepared in lyophilized form. With its fast turnaround time (3 weeks from expressed antigen to lyophilized probe) and low cost, the process has the potential to routinely overcome a significant bottleneck in diagnostics and research.

## Materials and Methods

### Reagents

Except where indicated, all chemicals were from Sigma-Aldrich (St. Louis, MO). All secondary reagents for immuno-cytometry were acquired through Molecular Probes (Invitrogen, Carlsbad, CA). Nickel-NTA sepharose for purification of expressed antigens was obtained from Qiagen.

The polyclonal rabbit anti-350 and anti-780 antisera were both generated by Cocalico Biologicals, Inc., Reamstown, PA, USA).

Proteins 780, 350, 142, and Jacob were biotinylated using either the Pierce EZ-Link Peg-Biotin or the Sulfo-NHS-LC-Biotin kit (Thermo Scientific, Rockford, IL) per manufacturer's instructions. The degree of biotinylation was quantified using the Pierce Biotin Quantitation (HABA) Assay (Thermo Scientific, Rockford, IL).

### Protein purification

The divergent region of *E. histolytica* Jacob protein (compared to *E. dispar* and *E. moshkovskii* proteins) was codon optimized (GeneArt, Invitrogen) and topo-cloned into the pET SUMO vector. The correct orientation of insert in pET SUMO vector was verified by DNA sequencing.

For the chromodomain proteins (EHI_000780, EHI_115350, and EHI_142000), DNA segments corresponding to the selected region of proteins were PCR amplified from the *E. histolytica* genomic DNA, digested with *Nde*I and *Bam*H1 restriction enzymes, gel-purified, and ligated to pET11a vector using the T4 DNA ligase. Top10 cells were then transformed using the pET11a vector containing the insert. Both pET SUMO vector and pET11a vectors contain sequences for 6 histidine residues N-terminal to the gene of interest, allowing each protein to be purified using the Ni-NTA column according to the manufacturer's instructions (Qiagen). The SUMO fusion protein was cleaved from the *Eh*Jacob with the treatment of SUMO protease (LifeSensors, Malvern, PA).

### Library selections

Selections were performed as described previously [1], [6], [7], [20]. Yeast were routinely grown in SDCAA broth. To induce expression of scFv on the yeast surface, yeast were grown in selective synthetic galactose/raffinose plus casamino acids deficient in histidine, uracil, and tryptophan (SG/RCAA) broth. For round 1 (R1) and round 2 (R2) of selection, 10^10^ yeast were incubated in 10 mL yeast wash buffer (YWB; PBS 0.5% BSA) containing 100 nM of each of the four biotinylated proteins separately. Yeast that bound protein were then labeled by incubation with 200 µL of either streptavidin-magnetic particles (R1) or anti-biotin-magnetic particles (R2). Following the second magnetic sort, the eluted yeast (R2 output) were incubated with 1 µg mL^−1^ SA-PE. Yeast were then sorted by FACS for the top 10% of PE-positive cells and expanded into 5 mL of SDCAA broth (sorted yeast comprised the R3 output. For round 4 (R4) of the selection, the R3a outputs were incubated with anti-c-myc MAb and goat-anti-mouse- fluorescein isothiocyanate (GaM-FITC) to confirm expression of scFv before again being incubated with 100 nM of their specific biotinylated antigen followed by SA-PE. Yeast were then sorted by FACS for FITC-positive and the top 10% of PE-positive signal. Antigen-binding yeast were grown on synthetic dextrose casamino acids minus His, Ura, Trp plate (-HUT media) for single clone analysis. For each antigen, we picked 24–48 clones and grew them in 96 deep well plates in 1 mL of SDCAA broth. Following induction in 1 mL SGRCAA, each clone was then tested by flow cytometry for binding to cognate antigen and for non-binding to all non-cognate antigens or the secondary reagents (SA-PE, FITC) themselves. Antigen-specific clones were then amplified by PCR, and the PCR amplicon was subjected to *Bst*N1 fingerprint analysis as described previously [6], [7]. In this method, the 850–1000 nucleotide PCR amplicons are digested with the restriction endonuclease *Bst*N1, usually resulting in 3–8 smaller fragments. The resulting fragment pattern, or fingerprint, after resolving the fragments by DNA gel electrophoresis is used to determine uniqueness. All apparently unique clones were then DNA sequenced to confirm uniqueness and confirmed clones were used in the study.

To determine affinities, we used a previously described flow cytometry assay which utilizes whole yeast [20], [21], [22], [23]. In this assay, yeast-displaying scFv were incubated with twofold serial dilutions of biotinylated antigens spanning 3.125–250 nM in concentration and antigen binding was determined by further staining with SA-PE. Samples were analyzed by flow cytometry, results graphed as a function of antigen-biotin concentration versus mean PE fluorescence. Affinities were then determined by using a nonlinear least squares fit of the curves as previously described [1], [21], [24]. This assay was used to measure the affinities of clones *780-23, Jacob-A11, 350-12*, and *350-E2* (binding to 350-biotin). The affinity of clone *350-E2* to unlabeled 350 was measured by using a modification of this assay, in which binding was detected by staining the yeast-antigen complexes with a rabbit anti-350 polyclonal serum and then further stained with goat-anti-rabbit PE. We did not test the affinity of clone *Jacob-A11* to unlabeled Jacob protein.

### Yeast lyophilization

Selected yeast clones were induced to express scFv and active antigen binding was confirmed as described above. Confirmed antigen-binding cells were lyophilized by harvesting 100 mL of SG/RCAA culture at an OD_600_ = 1.5–2 (1 OD_600_≈2×10^7^ yeast/mL). An aliquot of culture with ∼1×10^7^ total yeast was transferred to 1.5 mL freezer tubes and centrifuged for 1 min at 13000*g*. The supernatant was decanted, and the pellets were flash-frozen in liquid nitrogen for approximately 15 seconds. Without allowing the yeast to thaw, the tubes were loosely capped and transferred to a lyophilization beaker that had been previously cooled in a dry-ice/ethanol bath. The beaker was immediately capped, fit to the lyophilizer, and vacuum applied. Yeast were lyophilized overnight using a Millrock Technology (Kingston, NY) Model BT48A lyophilizer. Following lyophilization, tubes were tightly capped and stored at room temperature without desiccants or stabilizers.

### Specificity analysis

One aliquot of fresh, non-lyophilized *350-E2* yeast and one aliquot of lyophilized, rehydrated yeast (∼1×10^7^ yeast/aliquot) were incubated with anti-c-*myc* MAb followed by goat-anti-mouse FITC detection MAb to confirm expression of scFv. Following c-*myc* staining, the yeast were split into two sets of five equal aliquots. One set was incubated with 100 nM of biotinylated cognate antigen and three biotinylated non-cognate antigens (plus the no antigen control) for 1 hr at RT. Binding of biotinylated antigens was detected by addition of 100 µL of a 1/800 dilution of SA-PE for 30 minutes. The second set of aliquots received 100 nM of unlabeled 350, 142, Jacob, and 780 (plus the no antigen control). To detect binding of unlabeled antigens, the yeast were incubated with a 1/1000 dilution of rabbit anti-350 antiserum for 45 min at RT followed by a 1/800 dilution of goat-anti-rabbit-PE for 45 min at RT. Yeast were then analyzed by flow cytometry for scFv expression (*x*-axis; FITC fluorescence) and antigen binding (*y*-axis; PE fluorescence). The assay was performed three times, and each assay utilized an independent induction and lyophilization of yeast *350-E2*. The percent PE fluorescent yeast were determined by gating the percent PE-positive relative to the “no antigen” controls. All other yeast clones isolated in this study were analyzed for specificity in a similar fashion.

### Lower limit of detection

One aliquot of lyophilized *350-E2* yeast clone was resuspended in 1 mL YWB and split into 11 equal aliquots. Each aliquot was incubated with 2.5 mL of 350 antigen ranging from 20 nM to 0.04 nM in twofold serial dilutions (plus the 0 nM control). Following a 1 hour antigen incubation, yeast were washed to remove unbound antigen, and the bound 350 antigen was detected by incubating with a 1/2500 dilution of rabbit anti-350 antiserum. Final detection of bound 350 was achieved by incubation with 1/2500 dilution of goat-anti-rabbit-PE (GaR-PE) detection MAb for 1 hr. Samples were analyzed by flow cytometry and the percent of PE-positive yeast, relative to a no antigen control, were plotted versus the concentration of 350 antigen. The experiment was repeated three independent times, and each data point reflects the average percent PE-positive yeast plus and minus the standard deviation.

### Immunofluorescence microscopy assay

One aliquot of lyophilized *350-E2* yeast clone was reconstituted in 1 mL YWB and split into 8 equal aliquots. Each aliquot received 100 µL of 7 concentrations of cognate 350 or non-cognate 142 ranging from 500 nM to 0.69 nM in threefold serial dilutions (plus the 0 nM control). Bound antigens were detected by incubating with a 1/2500 dilution of rabbit anti-350 antiserum followed by 1/800 dilution of goat-anti-rabbit-FITC (GaR-FITC) detection MAb for 1 hr. Following three washes to remove unbound antigen, yeast pellets were resuspended in 100 µL, and 10 µL of yeast were spotted onto microscope slides and a coverslip applied. The yeast were imaged at 40× magnification using a Nikon TE2000U inverted microscope (Nikon Corporation, Tokyo, Japan) outfitted with phase contrast and DIC optics for visible imaging, a FITC filter, a high speed Cool Snap CCD camera, and a motorized stage for automated scanning. Image capture and analysis was performed using Metamorph software (Research Precision Instruments, Natick, MA).

To confirm staining of the perimeter of yeast, suggestive of scFv binding, the sample incubated with 500 nM antigen was examined at 90× magnification. For purposes of clarity, the image seen in Figure 4B has also been digitally zoomed approximately 3-fold to better discern the fluorescent staining pattern.

A second experiment was similarly performed to enumerate the approximate number of FITC-positive yeast relative to a concentration of cognate antigen. In this experiment, yeast were incubated with 11 concentrations of unlabeled 350 antigen ranging from 160 nM down to 0.16 nM in twofold serial dilutions. For each antigen concentration, a minimum of 3 independent fields were captured in visible mode followed immediately by the same three fields in FITC fluorescence mode. Total and FITC-fluorescent yeast were counted in each field using the Image J Software (Rasband, W.S., ImageJ, U.S. National Institutes of Health, Bethesda, Maryland, USA, imagej.nih.gov/ij/, 1997–2011.) Visible fields were background-subtracted with a 50-pixel rolling ball radius while sliding paraboloid was enabled and automated smoothing disabled. Automatic thresholding was done in grayscale with the default settings. Particles (cells) were analyzed from 64-∞ without circularity compensation, with edge exclusion and hole inclusion. Fluorescent (FITC) fields were background subtracted in the same fashion. Then each FITC image underwent a binary conversion allowing the watershed filter to be applied. After automatic triangle thresholding in grayscale using a dark background, the fluorescent particles were enumerated from 64-∞ without circularity compensation. In each field, percent fluorescence was calculated from the ratio of the number of fluorescent yeast (FITC) to the total number of yeast in the visible spectrum. The percentages were averaged and standard deviations calculated from at least three fields at a given antigen concentration.

### Lyophilization time course assay

To assess the stability of yeast-scFv following lyophilization, a fresh culture of yeast clone *780-23* was induced for expression of scFv and lyophilized in individual aliquots containing ∼1×10^7^ yeast. For the first two weeks, time points were taken twice weekly, and from then on time points were taken approximately once per week until little binding to cognate antigen was observed.

For each time point, one aliquot of lyophilized yeast was reconstituted in 1 mL YWB, stained for c-*myc* expression as described previously, and then split into 5 equal aliquots. The aliquots received 100 µL of 100 nM of one of either cognate 780-biotin or non-cognate Jacob-biotin antigen (plus a no antigen control). Following 45 min of antigen binding, yeast were washed 2× and bound antigen detected by incubation with a 1/800 dilution of SA-PE. Yeast were washed 3× and analyzed for FITC (*x*-axis) and PE (*y*-axis) fluorescence by flow cytometry. A gate was set to measure the total percent of PE-positive yeast relative to the “no antigen” control. The percent of antigen binding (percent PE-positive) was then plotted versus the day post lyophilization. The cognate antigen throughout this assay was 780-biotin, and the non-cognate antigen was Jacob-biotin. Starting at day 30, we included two other non-cognate antigens, 350-biotin and 142-biotin to better assess specificity. The assay was terminated on day 86 when binding to cognate antigen had dropped below 15%.

## Supporting Information

Figure S1 **Pre- and post-lyophilization activity determination with multiple yeast-scFv clones.** Yeast clones were tested for their ability to bind antigen following lyophilization. One hundred microliters of either fresh or lyophilized/rehydrated yeast were stained first for c-*myc*-FITC expression (*x*-axis) followed by binding to cognate antigen and to a non-cognate control (100 nM antigen concentration). The name of the clone is indicated on the far left column. The non-cognate and cognate antigens used to generate the flow cytometric plots are indicated on the right of the figure. Binding to antigen is indicated by an increase in PE fluorescence (*y*-axis). Clones *350-10* and *780-18* are *myc*-negative due to premature stop codons following the scFv gene. However, these clones appeared to bind antigen before and after lyophilization.
(TIF)

Figure S2 **Lyophilization time course assay with yeast-scFv clone** ***350-12*** **.** Three individual colonies of clone *350-12* were lyophilized separately on different days. Every 3 to 5 days, one aliquot of each was tested for binding to cognate antigen (100 nM 350-biotin) and to a non-cognate control antigen (100 nM 780-biotin) as described for Figure 5. The graph depicts the binding to the cognate or non-cognate antigens, as determined by percent of PE-positive yeast (*y*-axis), for each of the time periods tested (*x*-axis). Because of the staggered lyophilization dates, data were grouped into groups of 2 to 3 days, and each point represents the average and standard deviation for the day range indicated in the graph.
(TIF)
