# Supplementary figures and images for: Toward Low-Cost Affinity Reagents: Lyophilized Yeast-scFv Probes Specific for Pathogen Antigens
Source: PLoS One. 2012 Feb 20;7(2):e32042. doi: 10.1371/journal.pone.0032042 (PMC3282784; doi:10.1371/journal.pone.0032042)

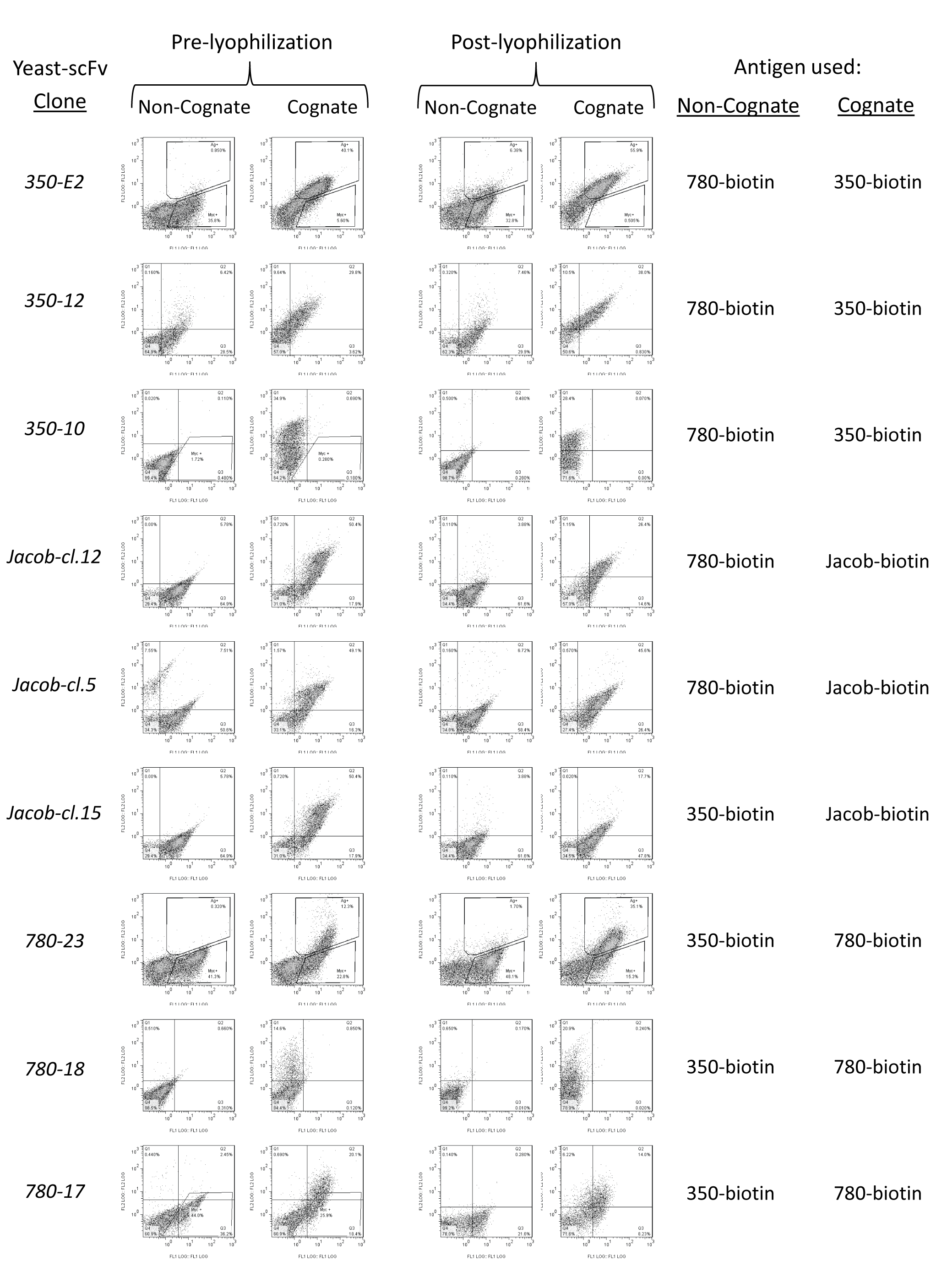

Supplement: Figure S1 — Pre- and post-lyophilization activity determination with multiple yeast-scFv clones. Yeast clones were tested for their ability to bind antigen following lyophilization. One hundred microliters of either fresh or lyophilized/rehydrated yeast were stained first for c-myc-FITC expression (x-axis) followed by binding to cognate antigen and to a non-cognate control (100 nM antigen concentration). The name of the clone is indicated on the far left column. The non-cognate and cognate antigens used to generate the flow cytometric plots are indicated on the right of the figure. Binding to antigen is indicated by an increase in PE fluorescence (y-axis). Clones 350-10 and 780-18 are myc-negative due to premature stop codons following the scFv gene. However, these clones appeared to bind antigen before and after lyophilization. (TIF) [file pone.0032042.s001.tif]

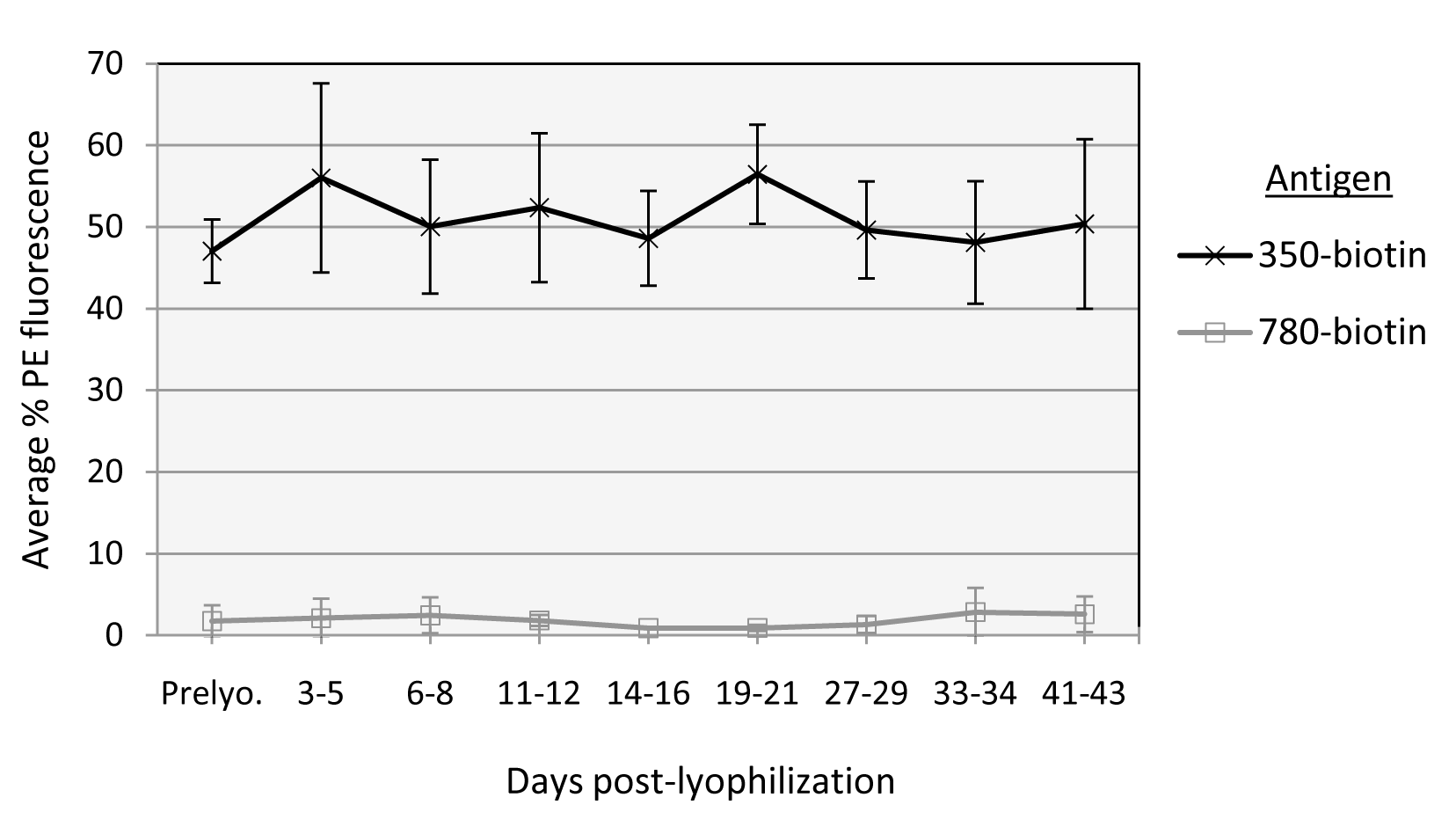

Supplement: Figure S2 — Lyophilization time course assay with yeast-scFv clone 350-12 . Three individual colonies of clone 350-12 were lyophilized separately on different days. Every 3 to 5 days, one aliquot of each was tested for binding to cognate antigen (100 nM 350-biotin) and to a non-cognate control antigen (100 nM 780-biotin) as described for Figure 5. The graph depicts the binding to the cognate or non-cognate antigens, as determined by percent of PE-positive yeast (y-axis), for each of the time periods tested (x-axis). Because of the staggered lyophilization dates, data were grouped into groups of 2 to 3 days, and each point represents the average and standard deviation for the day range indicated in the graph. (TIF) [file pone.0032042.s002.tif]
